# Crystal structure of flufenoxuron: a benzoyl­urea pesticide

**DOI:** 10.1107/S1600536814020649

**Published:** 2014-09-17

**Authors:** Youngeun Jeon, Gihaeng Kang, Sangjin Lee, Tae Ho Kim

**Affiliations:** a Department of Chemistry and Research Institute of Natural Sciences, Gyeongsang, National University, Jinju 660-701, Republic of Korea

**Keywords:** crystal structure, benzoyl­urea, pesticide, N—H⋯O hydrogen bonds, C—H⋯π inter­actions, π–π inter­actions

## Abstract

The title compound, C_21_H_11_ClF_6_N_2_O_3_ (systematic name: 1-{4-[2-chloro-4-(trifluoromethyl)phenoxy]-2-fluorophenyl}-3-(2,6-di­fluoro­benzo­yl)urea), is a benzoyl­urea pesticide. The dihedral angles between the central fluoro­benzene ring and the terminal di­fluoro­phenyl ring and chloro­phenyl ring system are 62.15 (5) and 88.03 (5)°, respectively. In the crystal, N—H⋯O hydrogen bonds link adjacent mol­ecules, forming *R*_2_^2^(8) inversion dimers that pack into loop chains along the *a-*axis direction by short F⋯F contacts [2.729 (2) Å]. In addition, the chains are linked by weak C—H⋯π and π–π inter­actions [inter-centroid distances = 3.661 (2) and 3.535 (12) Å], resulting in a three-dimensional architecture.

## Related literature   

For information on the toxicity and pesticidal properties of the title compound, see: Kamel *et al.* (2007 ▶); Salokhe *et al.* (2006 ▶). For a related crystal structure, see: Liu *et al.* (2008 ▶).
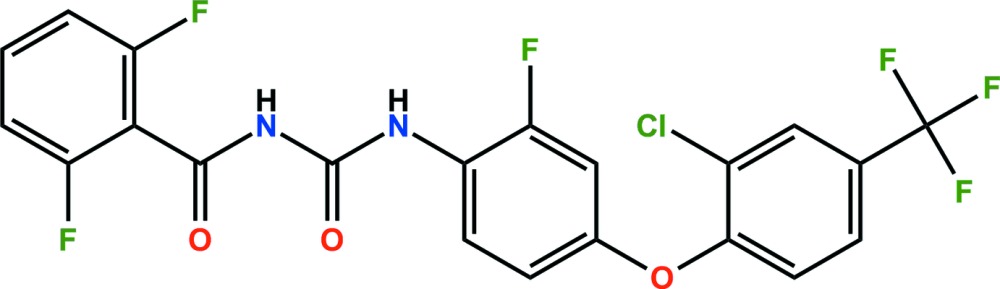


## Experimental   

### Crystal data  

C_21_H_11_ClF_6_N_2_O_3_
*M* *_r_* = 488.77
Triclinic, 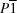

*a* = 10.017 (2) Å
*b* = 10.640 (2) Å
*c* = 10.677 (2) Å
α = 62.520 (9)°
β = 75.874 (8)°
γ = 81.831 (8)°
*V* = 978.4 (4) Å^3^
*Z* = 2
Mo *K*α radiation
μ = 0.28 mm^−1^
*T* = 173 K
0.35 × 0.30 × 0.28 mm

### Data collection  

Bruker APEXII CCD diffractometer
Absorption correction: multi-scan (*SADABS*; Bruker, 2009 ▶) *T*_min_ = 0.908, *T*_max_ = 0.926
14229 measured reflections
3822 independent reflections
3324 reflections with *I* > 2σ(*I*)
*R*_int_ = 0.023

### Refinement  

*R*[*F* ^2^ > 2σ(*F* ^2^)] = 0.038
*wR*(*F* ^2^) = 0.108
*S* = 1.07
3822 reflections
298 parameters
H-atom parameters constrained
Δρ_max_ = 0.27 e Å^−3^
Δρ_min_ = −0.30 e Å^−3^

### 

Data collection: *APEX2* (Bruker, 2009 ▶); cell refinement: *SAINT* (Bruker, 2009 ▶); data reduction: *SAINT*; program(s) used to solve structure: *SHELXTL* (Sheldrick, 2008 ▶); program(s) used to refine structure: *SHELXTL* ; molecular graphics: *DIAMOND* (Brandenburg, 2010 ▶); software used to prepare material for publication: *SHELXTL*.

## Supplementary Material

Crystal structure: contains datablock(s) global, I. DOI: 10.1107/S1600536814020649/su2781sup1.cif

Structure factors: contains datablock(s) I. DOI: 10.1107/S1600536814020649/su2781Isup2.hkl

Supporting information file. DOI: 10.1107/S1600536814020649/su2781Isup3.cml

**Fig. 1.**
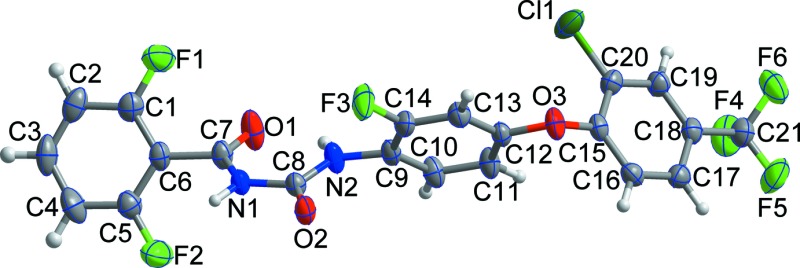
The molecular structure of the title molecule, with atom labelling. Displacement ellipsoids are drawn at the 50% probability level.

**Fig. 2.**
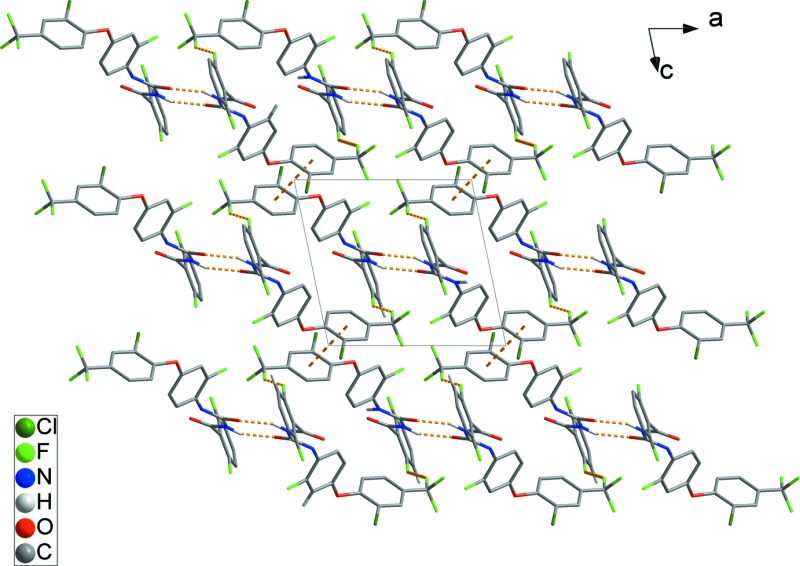
Crystal packing of the title compound viewed along the b axis. The N—H···O hydrogen bonds, weak π···π interactions, and short F···F contacts are shown as dashed lines (see Table 1 for details).

. DOI: 10.1107/S1600536814020649/su2781fig1.tif
The mol­ecular structure of the title mol­ecule, with atom labelling. Displacement ellipsoids are drawn at the 50% probability level.

b . DOI: 10.1107/S1600536814020649/su2781fig2.tif
Crystal packing of the title compound viewed along the *b* axis. The N—H⋯O hydrogen bonds, weak π⋯π inter­actions, and short F⋯F contacts are shown as dashed lines (see Table 1 for details).

CCDC reference: 1024205

Additional supporting information:  crystallographic information; 3D view; checkCIF report

## Figures and Tables

**Table 1:** Hydrogen-bond geometry (Å, °) *Cg*2 is the centroid of the C9–C14 ring.

*D*—H⋯*A*	*D*—H	H⋯*A*	*D*⋯*A*	*D*—H⋯*A*
N1—H1⋯O2^i^	0.88	1.97	2.8157 (17)	161
C2—H2*A*⋯*Cg*2^ii^	0.95	2.89	3.661 (2)	139

Symmetry codes: (i) 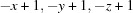; (ii) 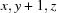.

## supplementary crystallographic information

### S1. Comment

Flufenoxuron is a benzoylurea pesticide and acts as an insect growth regulator and chitin synthesis inhibitor. It is used to control immature stages of insects and phytophagous mites on fruits and vegetables (Salokhe *et al.*, 2006; Kamel *et al.*, 2007) and its crystal structure is reported on herein.

In th e title compound, Fig. 1, the dihedral angles between the central fluorobenzene ring and the terminal difluorophenyl ring and chlorophenyl ring system are 62.15 (5) and 88.03 (5)°, respectively. All bond lengths and bond angles are normal and comparable to those observed in the crystal structure of a similar compound (Liu *et al.*, 2008).

In the crystal, Fig. 2, molecules are linked by a a pair of urea N—H···O hydrogen bonds (Table 1), forming inversion dimers with an *R*_2_^2^(8) ring motif. In addition, a short F···F contact [F2···F5^i^, 2.729 (2) Å] links the dimers into one-dimensional chains extending along [100]. In addition, a weak intermolecular C—H···π interaction [C2—H2A···*Cg*2^ii^, 3.611 (2) Å] and π–π interaction between the terminal chlorophenyl ring systems [*Cg*3···*Cg*3^iii^, 3.535 (12) Å] are present (*Cg*2 and *Cg*3 are the centroids of the C9-C14 and C15-C20 rings, respectively) [for symmetry codes: (i), -*x* + 1, -*y* + 1, -*z* + 1, (ii), *x*, *y* + 1, *z*, and (iii), -*x* + 2, -*y* + 1, -*z* + 2].

### S2. Experimental

The title compound was purchased from the Dr. Ehrenstorfer GmbH Company. Slow evaporation of a solution in CH_2_Cl_2_ gave single crystals suitable for X-ray analysis.

### S3. Refinement

All H-atoms were positioned geometrically and refined using a riding model: N—H = 0.88 Å, *U*_iso_ = 1.2*U*_eq_(C) C—H = 0.95 Å, with *U*_iso_ = 1.2*U*_eq_(N,C).

### Crystal data

**Table:**

C_21_H_11_ClF_6_N_2_O_3_	*Z* = 2
*M**_r_* = 488.77	*F*(000) = 492
Triclinic, *P*1	*D*_x_ = 1.659 Mg m^−^^3^
Hall symbol: -P 1	Mo *K*α radiation, λ = 0.71073 Å
*a* = 10.017 (2) Å	Cell parameters from 8341 reflections
*b* = 10.640 (2) Å	θ = 2.2–28.4°
*c* = 10.677 (2) Å	µ = 0.28 mm^−^^1^
α = 62.520 (9)°	*T* = 173 K
β = 75.874 (8)°	Block, colourless
γ = 81.831 (8)°	0.35 × 0.30 × 0.28 mm
*V* = 978.4 (4) Å^3^	

### Data collection

**Table:**

Bruker APEXII CCD diffractometer	3822 independent reflections
Radiation source: fine-focus sealed tube	3324 reflections with *I* > 2σ(*I*)
Graphite monochromator	*R*_int_ = 0.023
φ and ω scans	θ_max_ = 26.0°, θ_min_ = 2.1°
Absorption correction: multi-scan (*SADABS*; Bruker, 2009)	*h* = −12→12
*T*_min_ = 0.908, *T*_max_ = 0.926	*k* = −13→13
14229 measured reflections	*l* = −13→13

### Refinement

**Table:**

Refinement on *F*^2^	Primary atom site location: structure-invariant direct methods
Least-squares matrix: full	Secondary atom site location: difference Fourier map
*R*[*F*^2^ > 2σ(*F*^2^)] = 0.038	Hydrogen site location: inferred from neighbouring sites
*wR*(*F*^2^) = 0.108	H-atom parameters constrained
*S* = 1.07	*w* = 1/[σ^2^(*F*_o_^2^) + (0.0571*P*)^2^ + 0.2919*P*] where *P* = (*F*_o_^2^ + 2*F*_c_^2^)/3
3822 reflections	(Δ/σ)_max_ = 0.001
298 parameters	Δρ_max_ = 0.27 e Å^−^^3^
0 restraints	Δρ_min_ = −0.30 e Å^−^^3^

### Special details

**Table:**

Geometry. All e.s.d.'s (except the e.s.d. in the dihedral angle between two l.s. planes) are estimated using the full covariance matrix. The cell e.s.d.'s are taken into account individually in the estimation of e.s.d.'s in distances, angles and torsion angles; correlations between e.s.d.'s in cell parameters are only used when they are defined by crystal symmetry. An approximate (isotropic) treatment of cell e.s.d.'s is used for estimating e.s.d.'s involving l.s. planes.
Refinement. Refinement of *F*^2^ against ALL reflections. The weighted *R*-factor *wR* and goodness of fit *S* are based on *F*^2^, conventional *R*-factors *R* are based on *F*, with *F* set to zero for negative *F*^2^. The threshold expression of *F*^2^ > σ(*F*^2^) is used only for calculating *R*-factors(gt) *etc*. and is not relevant to the choice of reflections for refinement. *R*-factors based on *F*^2^ are statistically about twice as large as those based on *F*, and *R*- factors based on ALL data will be even larger.

### Fractional atomic coordinates and isotropic or equivalent isotropic displacement parameters (Å^2^)

**Table:**

	*x*	*y*	*z*	*U*_iso_*/*U*_eq_	
Cl1	1.05546 (6)	−0.22505 (6)	1.07307 (7)	0.06363 (18)	
F1	0.72246 (14)	0.77531 (13)	0.68591 (13)	0.0641 (3)	
F2	0.69987 (15)	0.81354 (13)	0.23893 (12)	0.0675 (4)	
F3	0.60803 (12)	0.16340 (12)	0.85938 (13)	0.0628 (3)	
F4	1.49311 (11)	−0.44174 (14)	0.79601 (15)	0.0679 (4)	
F5	1.39871 (13)	−0.60799 (15)	0.79606 (16)	0.0717 (4)	
F6	1.41608 (12)	−0.61650 (13)	0.99444 (13)	0.0641 (3)	
N1	0.66935 (13)	0.55105 (12)	0.51095 (14)	0.0308 (3)	
H1	0.5984	0.5943	0.4709	0.037*	
N2	0.78988 (14)	0.33879 (13)	0.61862 (17)	0.0392 (3)	
H2	0.8522	0.3912	0.6156	0.047*	
O1	0.86135 (14)	0.59246 (13)	0.56208 (18)	0.0583 (4)	
O2	0.59312 (11)	0.34257 (11)	0.55010 (13)	0.0374 (3)	
O3	0.87566 (11)	−0.24450 (11)	0.90357 (13)	0.0390 (3)	
C1	0.70453 (17)	0.85551 (18)	0.5500 (2)	0.0403 (4)	
C2	0.67730 (18)	0.99811 (19)	0.5047 (2)	0.0499 (5)	
H2A	0.6733	1.0404	0.5669	0.060*	
C3	0.6561 (2)	1.07764 (19)	0.3669 (3)	0.0555 (5)	
H3	0.6367	1.1766	0.3335	0.067*	
C4	0.6621 (2)	1.0175 (2)	0.2763 (2)	0.0560 (5)	
H4	0.6475	1.0735	0.1809	0.067*	
C5	0.68988 (18)	0.87424 (18)	0.32706 (19)	0.0419 (4)	
C6	0.71198 (15)	0.78873 (15)	0.46415 (18)	0.0339 (3)	
C7	0.75540 (16)	0.63483 (16)	0.51697 (18)	0.0355 (4)	
C8	0.68046 (15)	0.40302 (15)	0.56139 (16)	0.0296 (3)	
C9	0.81003 (16)	0.18930 (15)	0.68433 (18)	0.0332 (3)	
C10	0.92643 (16)	0.12655 (16)	0.63537 (19)	0.0384 (4)	
H10	0.9899	0.1833	0.5515	0.046*	
C11	0.95320 (16)	−0.01844 (17)	0.70618 (19)	0.0382 (4)	
H11	1.0345	−0.0608	0.6719	0.046*	
C12	0.86001 (16)	−0.09984 (15)	0.82683 (17)	0.0315 (3)	
C13	0.74046 (17)	−0.04027 (17)	0.87751 (17)	0.0369 (4)	
H13	0.6752	−0.0971	0.9593	0.044*	
C14	0.71953 (17)	0.10336 (17)	0.80564 (18)	0.0370 (4)	
C15	1.00506 (16)	−0.30610 (15)	0.88616 (16)	0.0324 (3)	
C16	1.03725 (17)	−0.37490 (16)	0.79989 (17)	0.0365 (4)	
H16	0.9738	−0.3717	0.7452	0.044*	
C17	1.16171 (17)	−0.44842 (17)	0.79308 (17)	0.0366 (4)	
H17	1.1843	−0.4958	0.7335	0.044*	
C18	1.25330 (16)	−0.45299 (16)	0.87284 (17)	0.0340 (3)	
C19	1.22220 (17)	−0.38360 (17)	0.95874 (18)	0.0372 (4)	
H19	1.2857	−0.3865	1.0132	0.045*	
C20	1.09787 (17)	−0.31014 (16)	0.96449 (18)	0.0368 (4)	
C21	1.38909 (18)	−0.53102 (19)	0.8653 (2)	0.0426 (4)	

### Atomic displacement parameters (Å^2^)

**Table:**

	*U*^11^	*U*^22^	*U*^33^	*U*^12^	*U*^13^	*U*^23^
Cl1	0.0639 (3)	0.0762 (4)	0.0827 (4)	0.0180 (3)	−0.0260 (3)	−0.0621 (3)
F1	0.0877 (9)	0.0590 (7)	0.0562 (7)	−0.0061 (6)	−0.0288 (6)	−0.0267 (6)
F2	0.1045 (10)	0.0538 (7)	0.0453 (6)	−0.0072 (7)	−0.0209 (7)	−0.0190 (5)
F3	0.0616 (7)	0.0458 (6)	0.0604 (7)	0.0227 (5)	0.0026 (6)	−0.0204 (5)
F4	0.0379 (6)	0.0673 (8)	0.0858 (9)	−0.0064 (5)	0.0052 (6)	−0.0309 (7)
F5	0.0624 (8)	0.0797 (9)	0.1068 (10)	0.0300 (7)	−0.0318 (7)	−0.0716 (8)
F6	0.0514 (7)	0.0652 (7)	0.0604 (7)	0.0206 (6)	−0.0210 (6)	−0.0168 (6)
N1	0.0298 (6)	0.0220 (6)	0.0409 (7)	0.0023 (5)	−0.0152 (5)	−0.0113 (5)
N2	0.0356 (7)	0.0221 (6)	0.0619 (9)	0.0018 (5)	−0.0261 (7)	−0.0132 (6)
O1	0.0441 (7)	0.0319 (6)	0.1053 (12)	0.0034 (5)	−0.0421 (8)	−0.0241 (7)
O2	0.0350 (6)	0.0266 (5)	0.0551 (7)	0.0012 (4)	−0.0212 (5)	−0.0168 (5)
O3	0.0319 (6)	0.0237 (5)	0.0490 (7)	0.0025 (4)	−0.0073 (5)	−0.0071 (5)
C1	0.0345 (8)	0.0365 (8)	0.0518 (10)	−0.0056 (7)	−0.0105 (7)	−0.0191 (8)
C2	0.0396 (9)	0.0396 (10)	0.0765 (14)	−0.0066 (8)	−0.0031 (9)	−0.0336 (10)
C3	0.0423 (10)	0.0263 (8)	0.0855 (15)	−0.0017 (7)	−0.0064 (10)	−0.0175 (9)
C4	0.0546 (12)	0.0350 (9)	0.0571 (12)	−0.0039 (8)	−0.0148 (9)	−0.0003 (9)
C5	0.0435 (9)	0.0342 (8)	0.0445 (9)	−0.0045 (7)	−0.0098 (8)	−0.0133 (7)
C6	0.0285 (7)	0.0256 (7)	0.0453 (9)	−0.0039 (6)	−0.0094 (7)	−0.0122 (7)
C7	0.0324 (8)	0.0265 (7)	0.0471 (9)	−0.0016 (6)	−0.0133 (7)	−0.0132 (7)
C8	0.0294 (7)	0.0242 (7)	0.0339 (7)	0.0005 (6)	−0.0088 (6)	−0.0110 (6)
C9	0.0333 (8)	0.0243 (7)	0.0448 (9)	0.0018 (6)	−0.0196 (7)	−0.0128 (7)
C10	0.0292 (8)	0.0287 (8)	0.0473 (9)	−0.0035 (6)	−0.0087 (7)	−0.0075 (7)
C11	0.0285 (8)	0.0289 (8)	0.0487 (9)	0.0026 (6)	−0.0056 (7)	−0.0122 (7)
C12	0.0331 (8)	0.0230 (7)	0.0380 (8)	0.0025 (6)	−0.0137 (6)	−0.0110 (6)
C13	0.0357 (8)	0.0327 (8)	0.0359 (8)	0.0018 (7)	−0.0055 (7)	−0.0115 (7)
C14	0.0368 (8)	0.0345 (8)	0.0410 (9)	0.0092 (7)	−0.0111 (7)	−0.0192 (7)
C15	0.0324 (8)	0.0219 (7)	0.0353 (8)	0.0018 (6)	−0.0083 (6)	−0.0065 (6)
C16	0.0411 (9)	0.0320 (8)	0.0347 (8)	−0.0004 (7)	−0.0149 (7)	−0.0102 (7)
C17	0.0427 (9)	0.0324 (8)	0.0348 (8)	−0.0006 (7)	−0.0069 (7)	−0.0156 (7)
C18	0.0351 (8)	0.0259 (7)	0.0352 (8)	−0.0006 (6)	−0.0058 (7)	−0.0095 (6)
C19	0.0351 (8)	0.0358 (8)	0.0441 (9)	0.0018 (7)	−0.0131 (7)	−0.0190 (7)
C20	0.0416 (9)	0.0299 (8)	0.0412 (9)	0.0003 (7)	−0.0088 (7)	−0.0179 (7)
C21	0.0379 (9)	0.0400 (9)	0.0488 (10)	0.0031 (7)	−0.0074 (8)	−0.0207 (8)

### Geometric parameters (Å, º)

**Table:**

Cl1—C20	1.7214 (17)	C4—C5	1.373 (3)
F1—C1	1.344 (2)	C4—H4	0.9500
F2—C5	1.345 (2)	C5—C6	1.375 (2)
F3—C14	1.3444 (19)	C6—C7	1.501 (2)
F4—C21	1.339 (2)	C9—C10	1.372 (2)
F5—C21	1.315 (2)	C9—C14	1.380 (2)
F6—C21	1.325 (2)	C10—C11	1.389 (2)
N1—C7	1.3566 (19)	C10—H10	0.9500
N1—C8	1.4070 (18)	C11—C12	1.376 (2)
N1—H1	0.8800	C11—H11	0.9500
N2—C8	1.331 (2)	C12—C13	1.383 (2)
N2—C9	1.4180 (19)	C13—C14	1.369 (2)
N2—H2	0.8800	C13—H13	0.9500
O1—C7	1.214 (2)	C15—C16	1.378 (2)
O2—C8	1.2152 (18)	C15—C20	1.379 (2)
O3—C12	1.3767 (18)	C16—C17	1.379 (2)
O3—C15	1.3776 (18)	C16—H16	0.9500
C1—C2	1.371 (2)	C17—C18	1.378 (2)
C1—C6	1.379 (2)	C17—H17	0.9500
C2—C3	1.370 (3)	C18—C19	1.380 (2)
C2—H2A	0.9500	C18—C21	1.494 (2)
C3—C4	1.371 (3)	C19—C20	1.377 (2)
C3—H3	0.9500	C19—H19	0.9500
			
C7—N1—C8	127.52 (12)	C12—C11—C10	118.92 (15)
C7—N1—H1	116.2	C12—C11—H11	120.5
C8—N1—H1	116.2	C10—C11—H11	120.5
C8—N2—C9	122.64 (13)	C11—C12—O3	123.56 (14)
C8—N2—H2	118.7	C11—C12—C13	121.33 (14)
C9—N2—H2	118.7	O3—C12—C13	115.09 (13)
C12—O3—C15	118.26 (12)	C14—C13—C12	117.68 (15)
F1—C1—C2	118.69 (17)	C14—C13—H13	121.2
F1—C1—C6	117.85 (15)	C12—C13—H13	121.2
C2—C1—C6	123.45 (17)	F3—C14—C13	118.10 (15)
C3—C2—C1	117.89 (18)	F3—C14—C9	118.80 (14)
C3—C2—H2A	121.1	C13—C14—C9	123.05 (15)
C1—C2—H2A	121.1	O3—C15—C16	119.66 (14)
C2—C3—C4	121.44 (17)	O3—C15—C20	120.24 (14)
C2—C3—H3	119.3	C16—C15—C20	119.85 (14)
C4—C3—H3	119.3	C15—C16—C17	119.88 (15)
C3—C4—C5	118.30 (19)	C15—C16—H16	120.1
C3—C4—H4	120.8	C17—C16—H16	120.1
C5—C4—H4	120.8	C18—C17—C16	119.86 (15)
F2—C5—C4	119.35 (17)	C18—C17—H17	120.1
F2—C5—C6	117.60 (15)	C16—C17—H17	120.1
C4—C5—C6	123.02 (18)	C17—C18—C19	120.59 (15)
C5—C6—C1	115.89 (15)	C17—C18—C21	120.41 (15)
C5—C6—C7	123.76 (15)	C19—C18—C21	118.99 (15)
C1—C6—C7	120.09 (15)	C20—C19—C18	119.12 (15)
O1—C7—N1	124.19 (14)	C20—C19—H19	120.4
O1—C7—C6	120.04 (14)	C18—C19—H19	120.4
N1—C7—C6	115.76 (13)	C19—C20—C15	120.69 (15)
O2—C8—N2	124.43 (14)	C19—C20—Cl1	120.01 (13)
O2—C8—N1	119.65 (13)	C15—C20—Cl1	119.28 (12)
N2—C8—N1	115.91 (12)	F5—C21—F6	107.35 (15)
C10—C9—C14	117.81 (14)	F5—C21—F4	106.12 (15)
C10—C9—N2	120.57 (14)	F6—C21—F4	105.84 (15)
C14—C9—N2	121.43 (14)	F5—C21—C18	112.98 (15)
C9—C10—C11	121.19 (15)	F6—C21—C18	112.64 (14)
C9—C10—H10	119.4	F4—C21—C18	111.43 (14)
C11—C10—H10	119.4		
			
F1—C1—C2—C3	−178.48 (16)	C15—O3—C12—C11	19.0 (2)
C6—C1—C2—C3	0.2 (3)	C15—O3—C12—C13	−162.55 (14)
C1—C2—C3—C4	−0.2 (3)	C11—C12—C13—C14	−1.7 (2)
C2—C3—C4—C5	0.2 (3)	O3—C12—C13—C14	179.82 (14)
C3—C4—C5—F2	−178.29 (18)	C12—C13—C14—F3	−175.87 (15)
C3—C4—C5—C6	−0.1 (3)	C12—C13—C14—C9	1.6 (3)
F2—C5—C6—C1	178.30 (15)	C10—C9—C14—F3	176.97 (15)
C4—C5—C6—C1	0.1 (3)	N2—C9—C14—F3	2.0 (2)
F2—C5—C6—C7	4.2 (2)	C10—C9—C14—C13	−0.5 (2)
C4—C5—C6—C7	−174.03 (16)	N2—C9—C14—C13	−175.52 (15)
F1—C1—C6—C5	178.56 (15)	C12—O3—C15—C16	−101.84 (17)
C2—C1—C6—C5	−0.1 (2)	C12—O3—C15—C20	83.96 (18)
F1—C1—C6—C7	−7.1 (2)	O3—C15—C16—C17	−173.73 (14)
C2—C1—C6—C7	174.21 (15)	C20—C15—C16—C17	0.5 (2)
C8—N1—C7—O1	3.9 (3)	C15—C16—C17—C18	0.1 (2)
C8—N1—C7—C6	−175.67 (14)	C16—C17—C18—C19	−0.6 (2)
C5—C6—C7—O1	121.8 (2)	C16—C17—C18—C21	−179.38 (15)
C1—C6—C7—O1	−52.1 (2)	C17—C18—C19—C20	0.4 (2)
C5—C6—C7—N1	−58.6 (2)	C21—C18—C19—C20	179.20 (15)
C1—C6—C7—N1	127.49 (16)	C18—C19—C20—C15	0.3 (2)
C9—N2—C8—O2	−5.5 (3)	C18—C19—C20—Cl1	178.96 (12)
C9—N2—C8—N1	174.92 (14)	O3—C15—C20—C19	173.50 (14)
C7—N1—C8—O2	178.80 (15)	C16—C15—C20—C19	−0.7 (2)
C7—N1—C8—N2	−1.6 (2)	O3—C15—C20—Cl1	−5.2 (2)
C8—N2—C9—C10	122.32 (18)	C16—C15—C20—Cl1	−179.39 (12)
C8—N2—C9—C14	−62.8 (2)	C17—C18—C21—F5	−9.8 (2)
C14—C9—C10—C11	−0.6 (2)	C19—C18—C21—F5	171.34 (15)
N2—C9—C10—C11	174.45 (15)	C17—C18—C21—F6	−131.67 (17)
C9—C10—C11—C12	0.6 (3)	C19—C18—C21—F6	49.5 (2)
C10—C11—C12—O3	179.01 (15)	C17—C18—C21—F4	109.55 (18)
C10—C11—C12—C13	0.6 (3)	C19—C18—C21—F4	−69.3 (2)

### Hydrogen-bond geometry (Å, º)

Cg2 is the centroid of the C9–C14 ring.

**Table:**

*D*—H···*A*	*D*—H	H···*A*	*D*···*A*	*D*—H···*A*
N1—H1···O2^i^	0.88	1.97	2.8157 (17)	161
C2—H2*A*···*Cg*2^ii^	0.95	2.89	3.661 (2)	139

Symmetry codes: (i) −*x*+1, −*y*+1, −*z*+1; (ii) *x*, *y*+1, *z*.
