# Supplementary figures and images for: Crystal structure of flufenoxuron: a benzoyl­urea pesticide
Source: Acta Crystallogr Sect E Struct Rep Online. 2014 Sep 17;70(Pt 10):o1110. doi: 10.1107/S1600536814020649 (PMC4257195; doi:10.1107/S1600536814020649)

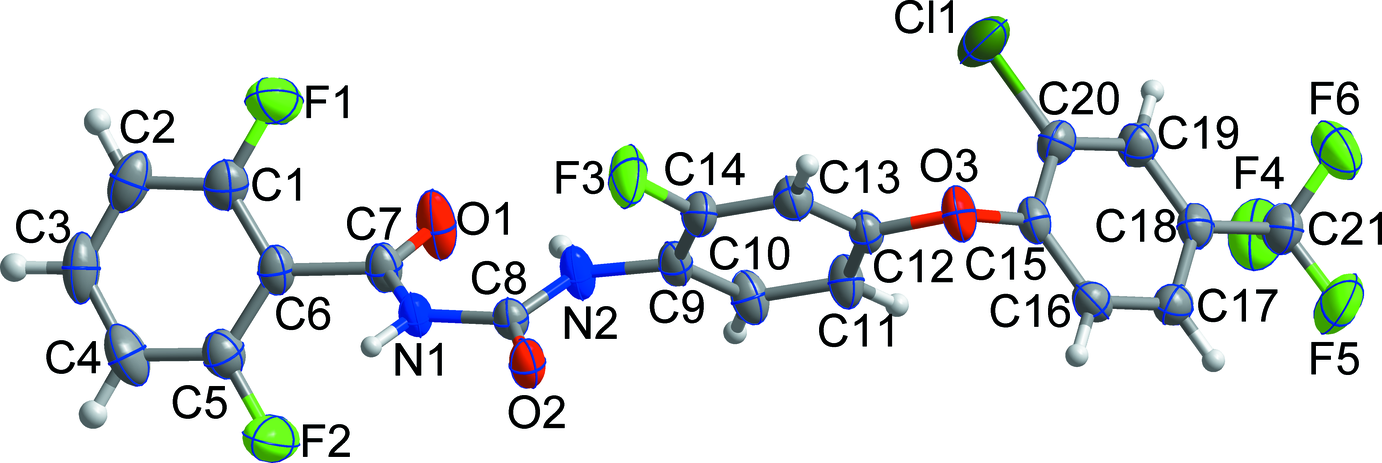

Supplement: Supplementary file 4 [file e-70-o1110-fig1.tif]

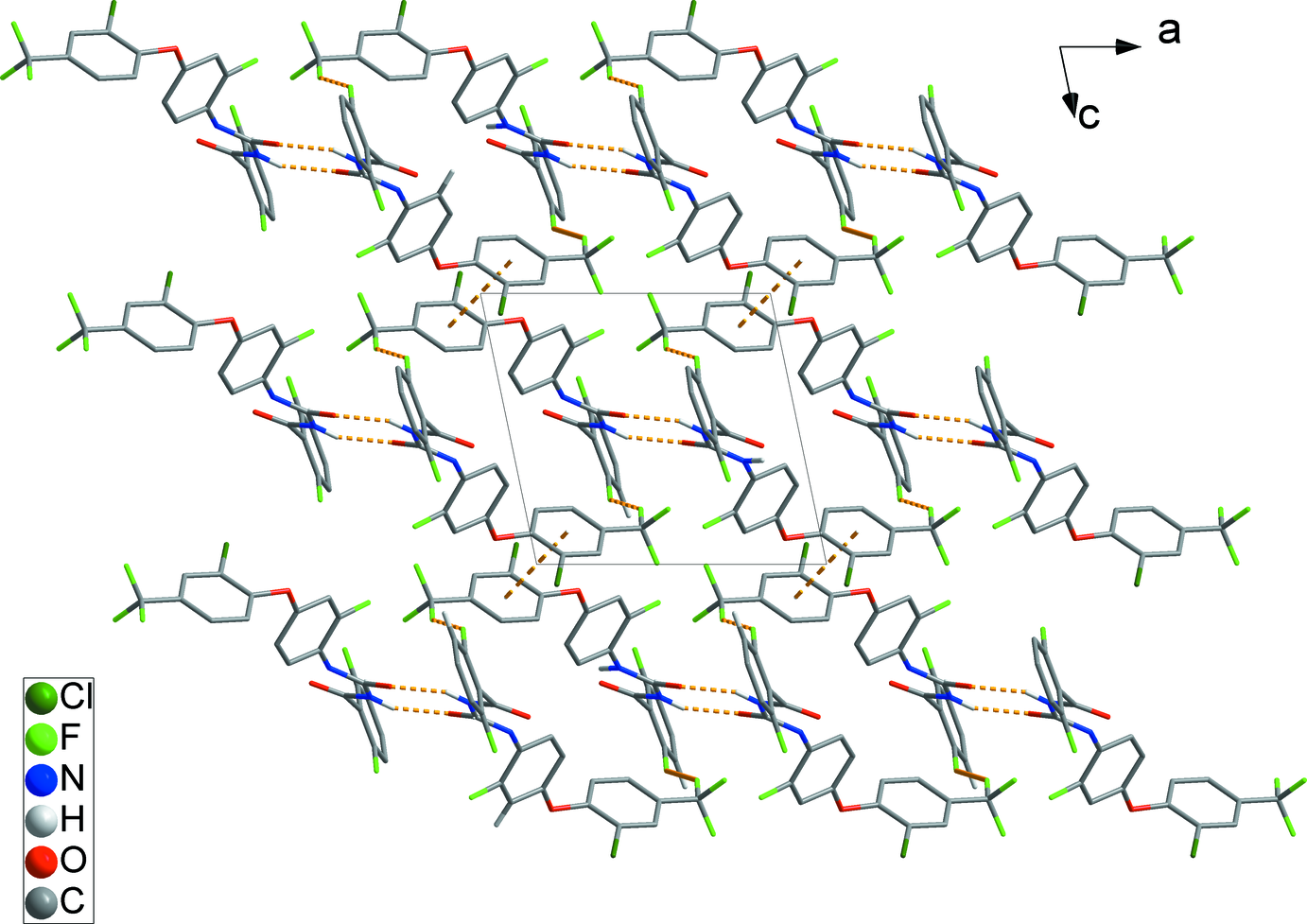

Supplement: Supplementary file 5 [file e-70-o1110-fig2.tif]
